# Enhanced Photocatalytic Activity of NaBH_4_ Reduced BiFeO_3_ Nanoparticles for Rhodamine B Decolorization

**DOI:** 10.3390/ma10101118

**Published:** 2017-09-22

**Authors:** Lijing Di, Hua Yang, Tao Xian, Xiujuan Chen

**Affiliations:** 1 State Key Laboratory of Advanced Processing and Recycling of Non-ferrous Metals, Lanzhou University of Technology, Lanzhou 730050, China; dlj0308@sina.com (L.D.); chenxj@lut.cn (X.C.); 2 College of Physics and Electronic Information Engineering, Qinghai Normal University, Xining 810008, China; xiantao1985@126.com

**Keywords:** BiFeO_3_, oxygen vacancies, photocatalysis

## Abstract

In this work, oxygen vacancies were introduced onto the surface of BiFeO_3_ nanoparticles by NaBH_4_ reduction method to yield oxygen-deficient BiFeO_3−x_ samples. Comprehensive analysis on the basis of high-resolution transmission electron microscopy (HRTEM) observation and X-ray photoelectron spectrum (XPS) confirms the existence of surface oxygen vacancies on the BiFeO_3−x_ nanoparticles. The photocatalytic activity of as-prepared BiFeO_3−x_ samples was evaluated by the decolorization of rhodamine B (RhB) under simulated sunlight irradiation. The experimental results indicate that the photocatalytic activity of samples is highly related to the NaBH_4_ reduction time, and the BiFeO_3−x_ sample reduced for 40 min exhibits the highest photocatalytic efficiency, which is much higher than that of pristine BiFeO_3_ nanoparticles. This can be explained by the fact that the surface oxygen vacancies act as photoinduced charges acceptors and adsorption sites suppress the recombination of photogenerated charges, leading to an increasing availability of photogenerated electrons and holes for photocatalytic reaction. In addition, the obtained BiFeO_3−x_ sample exhibits good photocatalytic reusability.

## 1. Introduction

Semiconductor photocatalysis has attracted tremendous interest because of its potential applications in solar energy conversion and environmental purification [1,2]. As a famous photocatalyst, TiO_2_ has been widely investigated due to its low cost and powerful photocatalytic capacity. However, TiO_2_ can be only excited under UV light irradiation, which accounts for 4% of the total solar energy. This limits the practical application of TiO_2_ as a photocatalyst. To make better use of solar energy that consists largely of visible light, it is essential to explore visible-light-driven photocatalysts [3,4,5].

BiFeO_3_ is an important perovskite-type oxide with outstanding multiferroic property. In addition to this excellent property, BiFeO_3_, as a narrow band gap semiconductor (~2.1 eV), exhibits visible light photocatalytic activity for the degradation of organic dyes and benzene [6,7,8,9,10]. However, its catalytic efficiency is not high enough for practical applications. It is well known that the catalytic activity of photocatalyst is closely related to various factors [1,2]. Among them, the effective separation of photogenerated electron-hole (e^−^-h^+^) pairs is very important in improving the photocatalytic activity. Up to now, many strategies have been used to modify BiFeO_3_, aiming to promote the separation of photogenerated charges [11,12,13,14,15].

Recently, it is reported that the introduction of oxygen vacancies on the surface of photocatalysts is demonstrated to be an efficient way to enhance their photocatalytic activities [16,17,18,19,20]. Generally, the surface oxygen vacancies can serve as the photogenerated charge traps and the adsorption sites, where the photoinduced charges can readily migrate to the adsorbed species. This process is expected to suppress the recombination of photogenerated electron-hole pairs, leading to an increased availability of electrons and holes for the photocatalytic reaction. Up to now, relatively little work has been devoted to the investigation of the photocatalytic property of BiFeO_3−x_ with surface oxygen vacancies. Most recently, Zhang and Wang et al. reported the preparation of BiFeO_3−x_ samples via the hydrogenation method and their enhanced photocatalytic performance [21,22]. However, this method involves harsh synthetic conditions, and furthermore expensive facilities are required. Compared with the hydrogenation method, the chemical reduction route has the main advantages of simplicity and low cost [23]. In this work, we develop a chemical reduction route, which is based on the NaBH_4_ reduction process, for the preparation of BiFeO_3−x_ with surface oxygen vacancies. The photocatalytic activity of products was evaluated by the decolorization of RhB under simulated sunlight irradiation, and the involved photocatalytic mechanism was proposed.

## 2. Experimental

BiFeO_3_ nanoparticles were synthesized by a polyacrylamide gel route as reported in the literature [7]. Stoichiometric amounts of Bi(NO_3_)_3_·5H_2_O and Fe(NO_3_)_3_·9H_2_O were dissolved into diluted HNO_3_ to form the transparent solution. Subsequently, the ethylenediamine-tetraacetic acid (EDTA) (in a 1.5:1 molar ratio with respect to the cations) was added into the above solution. After that, a certain amount of glucose was dissolved (20 g/100 mL). To the solution were added acrylamide and *N*,*N*′-methylene-bisacrylamide monomers with molar ratio of acrylamide/bisacrylamide (25/1), followed by adjusting the pH value to 3 by the addition of ammonia. The resulted solution was heated at 80 °C to initiate the polymerization reaction. The obtained gel was dried at 120 °C for 24 h, and then calcined at 600 °C for 3 h to obtain final BiFeO_3_ nanoparticles.

BiFeO_3−x_ samples were prepared via NaBH_4_ reduction method. 0.1 g BiFeO_3_ nanoparticles were dispersed into 20 mL NaBH_4_ solution (0.1 M) under magnetic stirring in ice-water bath. After reaction for a certain time, the product was separated by centrifugation, washed with distilled water and ethanol several times, and then dried in a vacuum drying oven at 60 °C for 4 h to obtain BiFeO_3−x_ samples. To study the effect of reduction time on the photocatalytic activity of BiFeO_3−x_ sample, a series of samples were prepared for different reduction times of 20, 40, and 60 min and termed as samples R20-BiFeO_3−x_, R40-BiFeO_3−x_, and R60-BiFeO_3−x_, respectively.

The photocatalytic activities of the samples were examined by the decolorization of rhodamine B (RhB) under simulated sunlight irradiation of a 300 W xenon lamp. In a typical photocatalytic process, the initial RhB concentration was 5 mg L^−1^ with a photocatalyst loading of 1 g L^−1^. Before irradiation, the suspension was magnetically stirred in the dark for 30 min to establish the adsorption-desorption equilibrium of RhB molecule on the surface of photocatalysts. Then the suspension was exposed to simulated sunlight irradiation under stirring. During the illumination, a small amount of reaction solution was taken every 1 h for measuring the concentration of RhB. Before the measurement, the suspension was centrifuged to separate the photocatalysts and obtain supernatant. The concentration of RhB was determined by detecting the absorbance of the supernatant at the wavelength 553 nm using an UV-VIS spectrophotometer. In order to evaluate the photocatalytic stability of samples, the recycling photocatalytic experimental was carried out. After the first cycle, the photocatalyst particles was collected by centrifugation and washed with water, and then dried in an oven. The recovered photocatalyst was added into the fresh RhB solution for the next cycle of the photocatalytic decolorization reaction under the same conditions.

The phase purity of products was examined by X-ray diffractometer (XRD). The morphology and structure of samples were observed using a transmission electron microscope (TEM). The UV-VIS diffuse reflectance spectra (DRS) of the samples were recorded by a UV-VIS spectrophotometer. The electron binding energies for the elements were measured by X-ray photoelectron spectrometer (XPS). The BET specific surface area of the sample is measured by the N_2_ adsorption-desorption technique on an ASAP2020M system (Micromeritics, Tristar II 3020, Norcross, GA, USA). The steady state photoluminescence spectra of samples were recorded by fluorescence lifetime and steady state spectroscopy (FLS920, Edinburgh Instrument, Livingston, Scotland, UK) with the excitation wavelength of ~350 nm.

## 3. Results and Discussion

**Figure 1:**
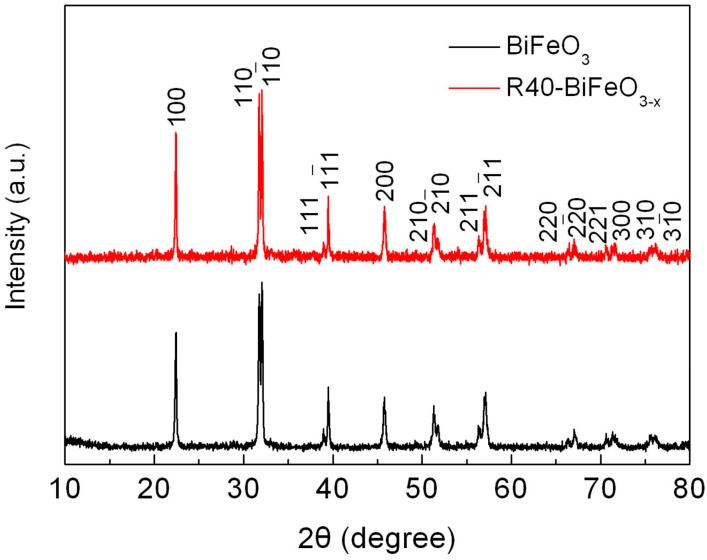
XRD patterns of BiFeO_3_ and R40-BiFeO_3−x_ samples.

Figure 1 shows the XRD patterns of BiFeO_3_ and R40-BiFeO_3−x_ samples. It can be seen that all the diffraction peaks of samples can be indexed to the rhombohedral structure of BiFeO_3_, and no traces of impurity phases are detected. This suggests that the NaBH_4_ reduction treatment has no remarkable influence on the phase purity of BiFeO_3_.

**Figure 2:**
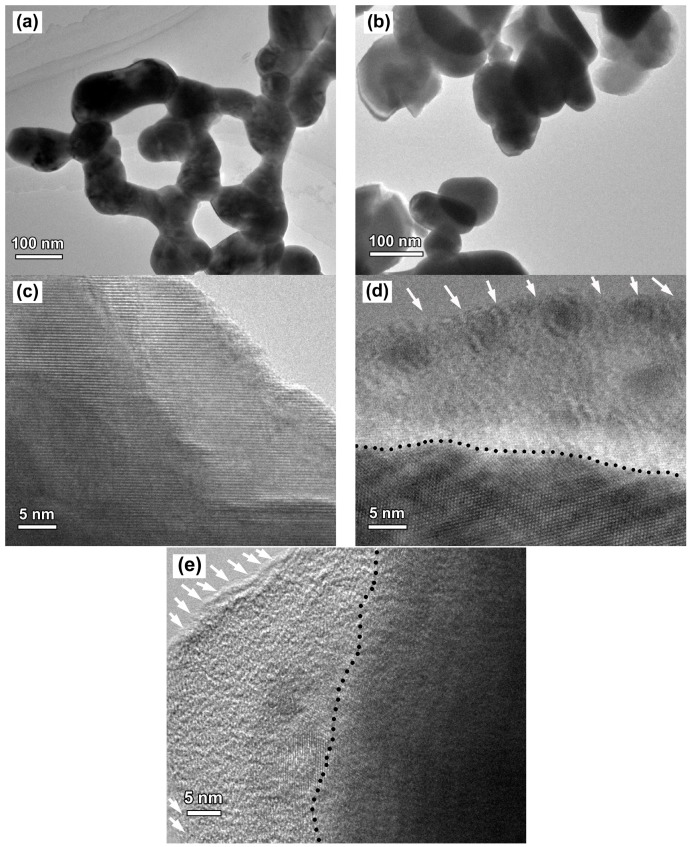
(**a**,**b**) TEM images of BiFeO_3_ and R40-BiFeO_3−x_ samples, respectively; (**c**–**e**) HRTEM images of BiFeO_3_, R40-BiFeO_3−x_, and R60-BiFeO_3−x_ samples, respectively; and (**d**,**e**), the black dash line shows the boundary between the crystalline core and the disordered layer (pointed out by white arrows).

Figure 2a,b show the TEM images of BiFeO_3_ and R40-BiFeO_3−x_ samples, respectively. Both the samples display sphere-like shape with size ranging from 80 nm to 110 nm, indicating that the morphology and size of BiFeO_3_ sample did not show obvious change after NaBH_4_ treatment. To further observe crystal structure of the products, the HRTEM images of BiFeO_3_, R40-BiFeO_3−x_, and R60-BiFeO_3−x_ samples are provided in Figure 2c–e, respectively. Figure 2c clearly presents the two-dimensional lattice fringes, revealing that the BiFeO_3_ nanoparticles are highly crystalline. For the R40-BiFeO_3−x_ sample, as shown in Figure 2d, the sample displays disordered edge with 15–20 nm thickness, while the inner part of sample is still well-crystallized. This suggests that the NaBH_4_ reduction leads to the creation of defect layer on the surface of BiFeO_3_ nanoparticles. In the case of R60-BiFeO_3−x_ sample, it is found that the thickness of disordered edge is about 20–30 nm (Figure 2e). This indicates that with increasing the reduction time, the thickness of the defect layer in the BiFeO_3_ exhibits an increasing trend.

**Figure 3:**
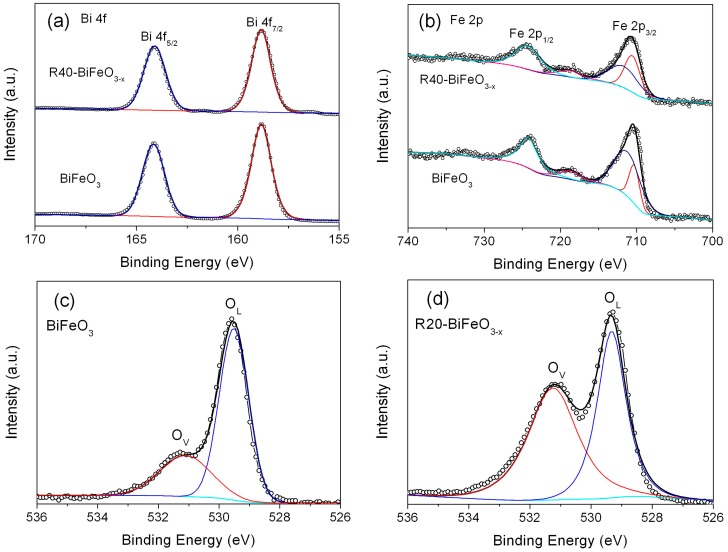

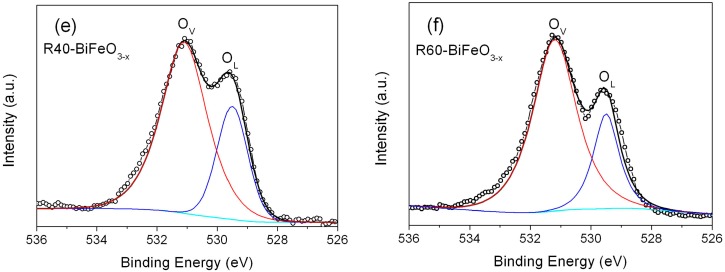
High-resolution XPS spectra of BiFeO_3_ and R40-BiFeO_3−x_ samples: (**a**) Bi 4f, (**b**) Fe 2p; O 1s high-resolution XPS spectra of BiFeO_3_ (**c**), R20-BiFeO_3−x_ (**d**), R40-BiFeO_3−x_ (**e**), and R60-BiFeO_3−x_ (**f**).

To further analyze the defect layer, the XPS detection is performed to investigate the surface chemical bonding of the BiFeO_3_ and reduced BiFeO_3_ samples. Figure 3a,b present the high-resolution XPS spectra for Bi 4f and Fe 2p in BiFeO_3_ and R40-BiFeO_3−x_, respectively. Two signals at binding energies of 164.1 eV and 159.2 eV for both samples correspond to the Bi 4f_5/2_ and Bi 4f_7/2_, respectively (Figure 3a), which are consistent with the chemical states of Bi^3+^ [24]. In Figure 3b, the intense peaks positioned at 724.1 eV in both samples are assigned to the 2p_1/2_ peaks of Fe^3+^. The other main peaks at 710.7 eV ascribing to Fe 2p_3/2_ are fitted into two peaks. These peaks situated on 711.5 eV and 710.2 eV are caused by the +3 and +2 oxidation state of Fe ion, respectively. Additionally, the satellite peaks centered at 718.3 eV for the two samples are observed, which is due to the multiple oxidation states of Fe. The XPS analysis of Fe element indicates the coexistence of Fe^2+^ and Fe^3+^ in the two samples. Furthermore, according to the analysis of the peak area in Figure 3b, the ratios of Fe^2+^ to Fe^3+^ in the BiFeO_3_ and R40-BiFeO_3−x_ are 23/77 and 48/52, respectively, which reveals that the concentration of Fe^2+^ in the sample is increased after the reduction treatment.

Figure 3c–f shows the high-resolution XPS spectra for O 1s in BiFeO_3_ and reduced BiFeO_3_ samples. One can see that the broad XPS peaks of O 1s can be divided into two peaks located at 531.1 eV and 529.6 eV, revealing two different kinds of O chemical state in the as-prepared samples. The peaks at 529.6 eV are ascribed to the lattice oxygen of BiFeO_3_ (named as O_L_), and the peaks at 531.1 eV are generally attributed to the chemisorbed oxygen caused by oxygen vacancies (named as O_v_) [18]. In nanosized BiFeO_3_, the long-range order of the lattice is commonly destroyed at the surface of sample, making the generation of oxygen vacancies. Compared to BiFeO_3_ nanoparticles, it is worth noting that the reduced BiFeO_3_ samples present a much higher peak at 531.1 eV compared with BiFeO_3_. Moreover, the analysis of the peak areas indicates that the concentration of the oxygen vacancies in the R20-BiFeO_3−x_, R40-BiFeO_3−x_, and R60-BiFeO_3−x_ are 0.53, 0.70, and 0.75, respectively. The results suggest that the concentration of oxygen vacancies increases with increasing the reduction time. It is well known that the detection depth of XPS is about 5 nm, therefore, the XPS spectrum information of reduced sample comes from the surface defect layer in the Figure 2d,e. Furthermore, it is generally accepted that the surface oxygen vacancies of oxides can destroy their surface lattice structure, and induce the generation of defect edge. Combined with the XPS and HRTEM analysis, it can be concluded that the defect layer on the NaBH_4_ reduced BiFeO_3_ nanoparticles is mainly attributed to the creation of a great amount of surface oxygen vacancies. On the other hand, the BET specific surface areas of the BiFeO_3_, R20-BiFeO_3−x_, R40-BiFeO_3−x_, and R60-BiFeO_3−x_ samples, measured by the N_2_ adsorption-desorption technique, are 6.52 m^2^/g, 6.49 m^2^/g, 6.55 m^2^/g, and 6.57 m^2^/g, respectively. This suggests that the surface area of BiFeO_3_ undergoes no obvious change after NaBH_4_ reduction.

**Figure 4:**
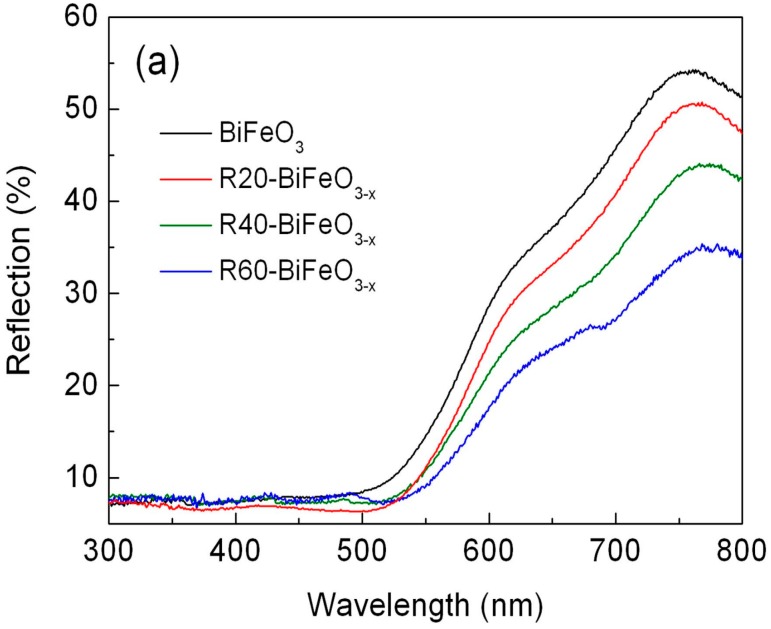

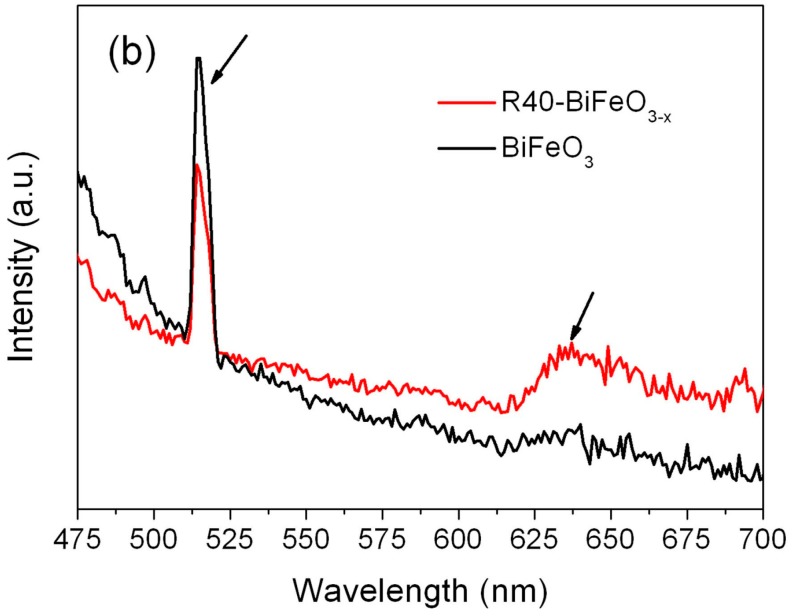
(**a**) UV-VIS diffuse reflectance spectra of BiFeO_3_ and BiFeO_3−x_ samples; and (**b**) the steady state photoluminescence spectra of BiFeO_3_ and R40-BiFeO_3−x_ samples.

Figure 4a shows the UV-VIS diffuse reflectance spectra of BiFeO_3_ and BiFeO_3−x_ samples. Compared with pristine BiFeO_3_ nanoparticles, the BiFeO_3−x_ samples show an enhanced light absorbance in the range of 550–800 nm. In order to exactly determine the band gap of samples, the steady state photoluminescence spectra of samples were carried out. As shown in Figure 4b, the strong and sharp emission peaks at 515 nm are observed for BiFeO_3_ and R40-BiFeO_3−x_. These emission peaks are attributed to the recombination of photogenerated charges between valence band (VB) and conduction band (CB), from which the band gap energy (*E*_g_) of BiFeO_3_ and R40-BiFeO_3−x_ is obtained to be 2.4 eV. In comparison to BiFeO_3_, it is worth noting that the R40-BiFeO_3−x_ exhibits an obvious emission peak centered at ~635 nm, which is considered to be the recombination of photoinduced charges between valence band and oxygen vacancy state. It is widely accepted that surface oxygen vacancies of semiconductor-based photocatalyst generally introduce an oxygen vacancy state within its forbidden gap [19].

**Figure 5:**
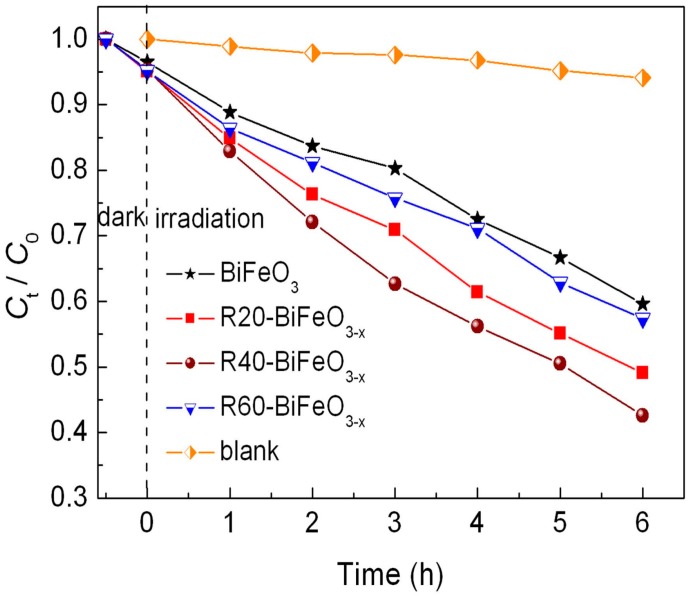
Photocatalytic degradation of RhB versus irradiation time in the presence of BiFeO_3_ and BiFeO_3−x_ samples, along with the blank and adsorption experiment results.

Figure 5 presents the photocatalytic decolorization of RhB under simulated sunlight irradiation as a function of reaction time in the presence of BiFeO_3_ and BiFeO_3−x_ samples. Before examining the photocatalytic properties, the blank and adsorption experiments are carried out. It is seen that no obvious decolorization of dye is observed in the absence of either photocatalysts or simulated sunlight, indicating that the impact of self-decolorization and adsorption on the photocatalytic effect can be ignored. In the presence of BiFeO_3_ nanoparticles, about 40% of RhB is decolored within 6 h. This illustrates that BiFeO_3_ nanoparticles process a moderate photocatalytic activity for the decolorization of RhB under simulated sunlight illumination. When the BiFeO_3_ sample is reduced by NaBH_4_, the reduction time exhibits an important influence on the photocatalytic performance of samples. With increasing the treatment time, the decolorization percentage of RhB is seen to gradually increase, from ~40% for *t*_treatment time_ = 0 min to ~57% for *t*_treatment time_ = 40 min. However, when the treatment time is further increased up to 60 min, the photocatalytic efficiency sharply decreases.

**Figure 6:**
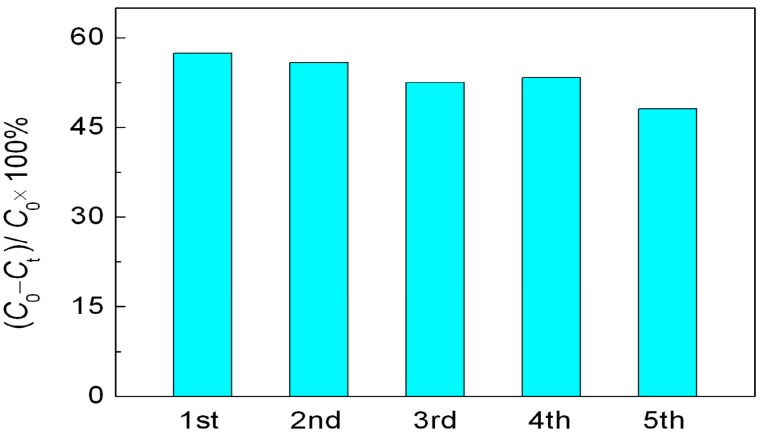
Cycling experiments in the photocatalytic degradation of RhB over R40-BiFeO_3−x_ sample for 6 h.

It is believed that the reusability of photocatalysts is crucial for their practical application. The recycling photocatalytic experiment is carried out under the same conditions to evaluate the stability of R40-BiFeO_3−x_ sample, as shown in Figure 6. After five recycles, R40-BiFeO_3−x_ sample maintains a high photocatalytic activity, indicating the stable photocatalytic activity of NaBH_4_ reduced BiFeO_3_ nanoparticles.

**Figure 7:**
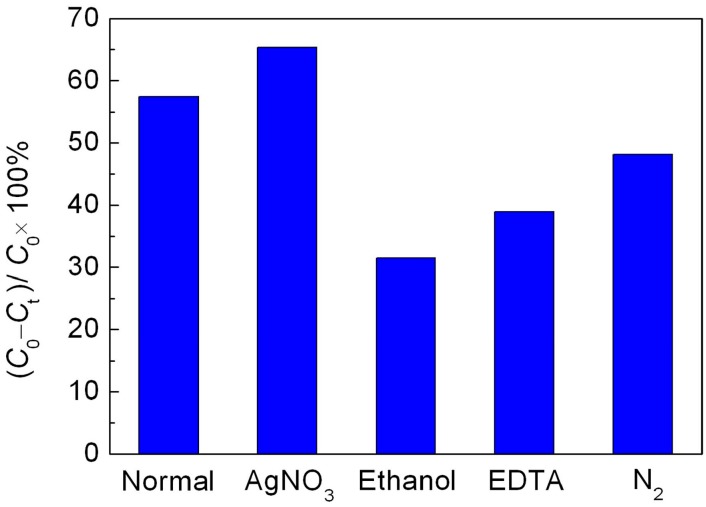
Effect of AgNO_3_, ethanol, EDTA and N_2_ on the photocatalytic decolorization of RhB over the R40-BiFeO_3−x_ sample.

To clarify the photocatalytic mechanism of NaBH_4_ reduced BiFeO_3_, the active species trapping experiments were performed. As shown in Figure 7, after adding AgNO_3_ (a scavenger of photogenerated electron (e^−^), 2 mM), the decolorization percentage of RhB is slightly increased compared to that without introduction of scavenger. This is mainly attributed to the efficient separation of photoinduced electron-hole pairs, resulting from the consumption of photogenerated electrons by AgNO_3_. When ethanol (a scavenger of hydroxyl radicals (•OH), 10% by volume) is introduced, the decolorization percentage of dye is significantly decreased, indicating that •OH is a main active species involved in this photocatalytic reaction. When ethylene diamine tetraacetic acid (EDTA, a scavenger of photogenerated holes (h^+^), 2 mM) is added, the decolorization efficiency of dye is also obviously suppressed, implying that h^+^ plays an important role in this photocatalysis. In addition to h^+^ and •OH, •O_2_ and H_2_O_2_ are considered to be another active species in the photocatalytic reaction. The effect of •O_2_ and H_2_O_2_, which are derived from the reaction between dissolved O_2_ and photogenerated electrons (e^−^), on the photocatalytic reaction can be detected by investigating the influence of N_2_ on the photocatalytic efficiency since the O_2_ molecules dissolved in reaction solution can be expelled from the solution by the N_2_-purging procedure. Upon bubbling with N_2_ (0.1 L/min), the decolorization percentage of dye undergoes a slight decrease, indicating relatively minor role of •O_2_ and/or H_2_O_2_ responsible for the dye decolorization.

**Figure 8:**
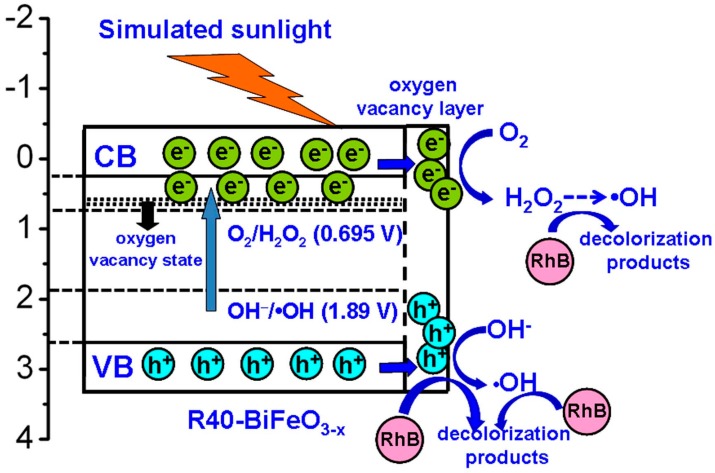
Schematic illustration of the possible promotion mechanism of surface oxygen vacancies on the simulated sunlight photocatalytic activity of reduced BiFeO_3_.

On the basis of above experimental results, a possible promotion mechanism of surface oxygen vacancies on the simulated sunlight photocatalytic activity of R40-BiFeO_3−x_ is proposed, as shown in Figure 8. Under the simulated sunlight irradiation, BiFeO_3_ is excited to generate photoexcited electron-hole pairs (Equation (3)). Unfortunately, the recombination rate of the photogenerated carries is high, leading to a moderate photocatalytic activity of BiFeO_3_. When oxygen vacancies are introduced onto the surface of BiFeO_3_ sample after NaBH_4_ reduction, an oxygen vacancy state appears in the forbidden gap of the BiFeO_3_, resulting in the electron transition from the valence band to oxygen vacancy state. This is beneficial to extend the light response region of BiFeO_3_. On the other hand, it is noted that the surface oxygen vacancies, which are excellent charge carrier acceptors and adsorption sites, can readily capture the photogenerated electron and promote the transfer of photoinduced charges to adsorbed species [20]. Consequently, the recombination of photogenerated charges can be suppressed, which results in an increasing availability of photogenerated electrons and holes participating in the photocatalytic redox reactions. However, it can be seen from Figure 5 that when the NaBH_4_ treatment time reaches 60 min, the photocatalytic efficiency exhibits an obvious decrease. The main reason is that excessive NaBH_4_ reduction is more likely to induce bulk oxygen vacancies. These bulk defects may serve as the new recombination centers for photogenerated electron-hole pairs and will lead to the reduction of photocatalytic efficiency.

To further investigate the photocatalytic redox reactions of photogenerated carriers, it is necessary to determine the energy-band potentials of photocatalysts. The valence band (VB) and conduction band (CB) potentials of the R40-BiFeO_3−x_ can be calculated according to the following equations:

*E*_VB_ = X − *E*^e^ + 0.5*E*_g_ (1)

*E*_CB_ = X − *E*^e^ − 0.5*E*_g_ (2)

where X and *E*^e^ are the absolute electronegativity of materials (defined as the arithmetic mean of the electron affinity and the first ionization of the constituent atoms) and energy of free electrons on the hydrogen scale (~4.5 eV), respectively. *E*_g_ is the band gap value of the R40-BiFeO_3−x_. The X value of BiFeO_3_ is calculated to be 5.93 eV based on the data reported in literatures [25,26]. As a result, the VB potential of R40-BiFeO_3−x_ is estimated to be 2.63 V vs. NHE, and the CB potential of R40-BiFeO_3−x_ is calculated to be 0.23 V vs. NHE. It can be seen that the VB potential of R40-BiFeO_3−x_ is positive to the redox potential of OH^−^/•OH (+1.89 V vs. NHE), indicating that photogenerated h^+^ can oxidize OH^−^ to form •OH (Equation (4)). On the other hand, the CB potential of R40-BiFeO_3−x_ is positive to the redox potential of O_2_/•O_2_ (−0.13 V vs. NHE), but negative to that of O_2_/H_2_O_2_ (+0.695 vs. NHE). This suggests that the photogenerated e^−^ can reduce O_2_ to generate H_2_O_2_ instead of •O_2_ (Equation (5)). Furthermore, H_2_O_2_ can undergoes a series of reactions to generate •OH radicals (Equation (6)). As a result, it is inferred that the h^+^, •OH, and H_2_O_2_ work together for the decolorization of RhB in the present photocatalytic reaction (Equation (7)), which is consistent with the trapping experimental results (Figure 7).

BiFeO_3_ + *hν* → BiFeO_3_ (e^−^ + h^+^) (3)

h^+^ + OH^−^ → •OH (4)

O_2_ + 2H^+^ + 2e^−^ → H_2_O_2_ (5)

H_2_O_2_ + *hν* → 2•OH (6)

h^+^, •OH or H_2_O_2_ + RhB → decolorization products (7)

## 4. Conclusions

The BiFeO_3−x_ nanoparticles with surface oxygen vacancies were successfully synthesized by a polyacrylamide gel method and subsequently reduced by NaBH_4_. Analysis results from HRTEM observations and XPS spectra reveal that the surface oxygen vacancies are introduced on the BiFeO_3−x_ nanoparticles. The photocatalytic experiments indicate that the photocatalytic performance of BiFeO_3−x_ nanoparticles for the decolorization of RhB under simulated sunlight irradiation depends highly on the NaBH_4_ reduction time. The BiFeO_3−x_ sample reduced for 40 min possesses the highest photocatalytic activity, which is much higher than that of pristine BiFeO_3_ nanoparticles. This can be attributed to the enhanced photogenerated charge separation and transport caused by surface oxygen vacancies, resulting in an increasing availability of photogenerated electrons and holes participating in the photocatalytic reactions. Moverover, the BiFeO_3−x_ nanoparticles exhibits good stability during the recycling photocatalytic experiment.

## References

[1] Fox M.A., Dulay M.T. (1993). Heterogeneous photocatalysis. Chem. Rev..

[2] Kudo A., Miseki Y. (2009). Heterogeneous photocatalyst materials for water splitting. Chem. Soc. Rev..

[3] Zhao D.Q., Wang W.W., Zong W.J., Xiong S.M., Zhang Q., Ji F.Y., Xu X. (2017). Synthesis of Bi_2_S_3_/BiVO_4_ heterojunction with a one-step hydrothermal method based on pH control and the evaluation of visible-light photocatalytic performance. Materials.

[4] Chiang T.H., Chen T.-M. (2017). Photocatalytic water splitting for O_2_ production under visible light irradiation using NdVO_4_-V_2_O_5_ hybrid powders. Materials.

[5] Bai X., Wang L., Zong R., Zhu Y. (2013). Photocatalytic activity enhanced via g-C_3_N_4_ nanoplates to nanorods. J. Phys. Chem. C.

[6] Gao F., Chen X.Y., Yin K.B., Dong S.A., Ren Z.F., Yuan F., Yu T., Zou Z.G., Liu J.M. (2007). Visible-light photocatalytic properties of weak magnetic BiFeO_3_ nanoparticles. Adv. Mater..

[7] Xian T., Yang H., Dai J.F., Wei Z.Q., Ma J.Y., Feng W.J. (2011). Photocatalytic properties of BiFeO_3_ nanoparticles with different sizes. Mater. Lett..

[8] Bharathkumar S., Sakar M., Balakumar S. (2016). Experimental evidence for the carrier transportation enhanced visible light driven photocatalytic process in bismuth ferrite (BiFeO_3_) one-dimensional fiber nanostructures. J. Phys. Chem. C.

[9] Sze-Mun L., Jin-Chung S., Abdul Rahman M. (2017). A newly emerging visible light-responsive BiFeO_3_ perovskite for photocatalytic applications: A mini review. Mater. Res. Bull..

[10] Bai X., Wei J., Tian B., Liu Y., Reiss T., Guiblin N., Gemeiner P., Dkhil B., Infante I.C. (2016). Size Effect on optical and photocatalytic properties in BiFeO_3_ nanoparticles. J. Phys. Chem. C.

[11] Navjot, Alexandr T., Singh L.G. (2017). Plasmonic enhanced photocatalytic activity of Ag nanospheres decorated BiFeO_3_ nanoparticles. Catal. Lett..

[12] Di L.J., Yang H., Hu G., Xian T., Ma J.Y., Jiang J.L., Li R.S., Wei Z.Q. (2014). Enhanced photocatalytic activity of BiFeO_3_ particles by surface decoration with Ag nanoparticles. J. Mater. Sci. Mater. Electron..

[13] Irfan S., Li L.L., Saleemi A.S., Nan C.W. (2017). Enhanced photocatalytic activity of La^3+^ and Se^4+^ co-doped bismuth ferrite nanostructures. J. Mater. Chem. A.

[14] Fan T., Chen C., Tang Z. (2016). Hydrothermal synthesis of novel BiFeO_3_/BiVO_4_ heterojunctions with enhanced photocatalytic activities under visible light irradiation. RSC Adv..

[15] Dhanalakshmi R., Muneeswaran M., Shalini K., Giridharan N.V. (2016). Enhanced photocatalytic activity of La-substituted BiFeO_3_ nanostructures on the degradation of phenol red. Mater. Lett..

[16] Chen X., Liu L., Yu P.Y., Mao S.S. (2011). Increasing solar absorption for photocatalysis with black hydrogenated titanium dioxide nanocrystals. Science.

[17] Tan H., Zhao Z., Zhu W.-B., Coker E.N., Li B., Zheng M., Yu W., Fan H., Sun Z. (2014). Oxygen vacancy enhanced photocatalytic activity of pervoskite SrTiO_3_. ACS Appl. Mater. Interfaces.

[18] Zhang X., Chen Z. (2015). Enhanced photoelectrochemical performance of the hierarchical micro/nano-structured TiO_2_ mesoporous spheres with oxygen vacancies via hydrogenation. RSC Adv..

[19] Zou X., Liu J., Su J., Zuo F., Chen J., Feng P. (2013). Facile Synthesis of thermal- and photostable titania with paramagnetic oxygen vacancies for visible-light photocatalysis. Chem. Eur. J..

[20] Pan X., Yang M.-Q., Fu X., Zhang N., Xu Y.-J. (2013). Defective TiO_2_ with oxygen vacancies: Synthesis, properties and photocatalytic applications. Nanoscale.

[21] Zhang C., Li Y., Chu M., Rong N., Xiao P., Zhang Y. (2016). Hydrogen-treated BiFeO_3_ nanoparticles with enhanced photoelectrochemical performance. RSC Adv..

[22] Wang S., Chen D., Niu F., Zhang N., Qin L., Huang Y. (2016). Hydrogenation-induced surface oxygen vacancies in BiFeO_3_ nanoparticles for enhanced visible light photocatalytic performance. J. Alloys Compd..

[23] Kang Q., Cao J., Zhang Y., Liu L., Xu H., Ye J. (2013). Reduced TiO_2_ nanotube arrays for photoelectrochemical water splitting. J. Mater. Chem. A.

[24] Wang X., Lin Y., Ding X., Jiang J. (2011). Enhanced visible-light-response photocatalytic activity of bismuth ferrite nanoparticles. J. Alloys Compd..

[25] Hotop H., Lineberger W.C. (1975). Binding energies in atomic negative ions. J. Phys. Chem. Ref. Data.

[26] Andersen T., Haugen H.K., Hotop H. (1999). Binding energies in atomic negative ions: III. J. Phys. Chem. Ref. Data.

